# Supplementary material for: Anti-diuretic hormone ITP signals via a guanylate cyclase receptor to modulate systemic homeostasis in Drosophila
Source: eLife. 2025 Nov 12;13:RP97043. doi: 10.7554/eLife.97043 (PMC12611267; doi:10.7554/eLife.97043)
Supplement: Supplementary file 1. [file elife-97043-supp1.docx]

Supplementary File 1: Fly strains used in this study

| **Fly strain** | **Stock number / Reference** |
| --- | --- |
| *ITP-RC-GAL4* | (Deng *et al.*, 2019) |
| *ITP-RD-GAL4* | BDSC# 84702 |
| *yolk-GAL4* | BDSC# 58814 |
| *Uro-GAL4* | (Halberg *et al.*, 2016) |
| *Uro-GAL4; UAS-dicer* | Dr. Kenneth Halberg |
| *c724-GAL4* | (Feingold *et al.*, 2019) |
| *CCAP-GAL4* | BDSC# 25685 |
| *Gyc76C-GAL4* | (Kondo *et al.*, 2020) |
| *Gyc32E-GAL4* | BDSC# 81160 |
| *Lkr-GAL4* | (Zandawala *et al.*, 2018) |
| *PK2-R1-GAL4* | BDSC# 84686 |
| *CG30340-GAL4* | BDSC# 84611 |
| *TkR99D-GAL4* | BDSC# 76208 |
| *UAS-Gyc76C-RNAi #1* | VDRC# 106525 |
| *UAS-Gyc76C-RNAi #2* | BDSC# 57315 |
| *UAS-ITP-RNAi* | VDRC# 330029 |
| *UAS-ITPa* | (Hermann-Luibl *et al.*, 2014) |
| *UAS-myr::tdTomato;2xLexAop-GFP;UASCaLexA,LexAopGFP/TM6B, Tb (UAS-CaLexA)* | Dr. Kenneth Halberg |
| *﻿JFRC81-10xUAS-IVS-Syn21-GFP-p10 (UAS-JFRC81GFP)* | (Pfeiffer *et al.*, 2012) |
| *﻿JFRC29-10xUAS-IVS-myr::GFP-p10 (UAS-JFRC29GFP)* | (Pfeiffer *et al.*, 2012) |
| *UAS-nls-mCherry* | BDSC# 38425 |
| *Tubulin-GAL80[ts]* | BDSC# 7017 |
| *w^1118^* | BDSC# 5905 |
| *UAS-Luciferase-RNAi* | BDSC# 31603 |
| *VDRC RNAi control* | VDRC# 60000 |

**Supplemental references**

Deng, B., Q. Li, X. Liu, Y. Cao, B. Li, Y. Qian, R. Xu, R. Mao, E. Zhou, W. Zhang, J. Huang and Y. Rao (2019). Chemoconnectomics: Mapping Chemical Transmission in *Drosophila*. *Neuron* **101**(5): 876-893 e874.

Feingold, D., L. Knogler, T. Starc, P. Drapeau, M. J. O'Donnell, L. A. Nilson and J. A. Dent (2019). secCl is a cys-loop ion channel necessary for the chloride conductance that mediates hormone-induced fluid secretion in *Drosophila*. *Sci Rep* **9**(1): 7464.

Halberg, K. A., S. M. Rainey, I. R. Veland, H. Neuert, A. J. Dornan, C. Klambt, S. A. Davies and J. A. Dow (2016). The cell adhesion molecule Fasciclin2 regulates brush border length and organization in *Drosophila* renal tubules. *Nat Commun* **7**: 11266.

Hermann-Luibl, C., T. Yoshii, P. R. Senthilan, H. Dircksen and C. Helfrich-Förster (2014). The ion transport peptide is a new functional clock neuropeptide in the fruit fly *Drosophila melanogaster*. *Journal of Neuroscience* **34**(29): 9522-9536.

Kondo, S., T. Takahashi, N. Yamagata, Y. Imanishi, H. Katow, S. Hiramatsu, K. Lynn, A. Abe, A. Kumaraswamy and H. Tanimoto (2020). Neurochemical Organization of the *Drosophila* Brain Visualized by Endogenously Tagged Neurotransmitter Receptors. *Cell Rep* **30**(1): 284-297 e285.

Pfeiffer, B. D., J. W. Truman and G. M. Rubin (2012). Using translational enhancers to increase transgene expression in *Drosophila*. *Proc Natl Acad Sci U S A* **109**(17): 6626-6631.

Zandawala, M., M. E. Yurgel, M. J. Texada, S. Liao, K. F. Rewitz, A. C. Keene and D. R. Nässel (2018). Modulation of *Drosophila* post-feeding physiology and behavior by the neuropeptide leucokinin. *PLoS Genet* **14**(11): e1007767.
