# Supplementary material for: Anti-diuretic hormone ITP signals via a guanylate cyclase receptor to modulate systemic homeostasis in Drosophila
Source: eLife. 2025 Nov 12;13:RP97043. doi: 10.7554/eLife.97043 (PMC12611267; doi:10.7554/eLife.97043)
Supplement: Supplementary file 2. [file elife-97043-supp2.docx]

Supplementary File 2: Antibodies used for immunohistochemistry

| **Antibody** | **Dilution** | **Source / Reference** |
| --- | --- | --- |
| ***Primary antibodies*** | | |
| chicken anti-GFP | 1:1000 | Abcam, RRID: AB_300798 |
| mouse anti-GFP | 1:1000 | Thermo Fisher Scientific |
| goat anti-GFP | 1:1000 | Rockland immunochemicals |
| guinea pig anti-GFP | 1:1500 | Synaptic systems (#132005) |
| rat anti-mCherry (16D7) | 1:1000 | Thermo Fisher Scientific |
| rabbit anti-ITPa | 1:5000 | Dr. Heinrich Dircksen (Hermann-Luibl *et al.*, 2014) |
| guinea pig anti-ITPa | 1:5000 | Dr. Heinrich Dircksen |
| rabbit anti-DH_31_ | 1:1000 | Dr. Jan Veenstra (Park *et al.*, 2008) |
| anti-*Diploptera punctata* Ast-A7 | 1:2000 | Dr. Christian Wegener |
| rabbit anti-NPF (to label Tv neurons) | 1:1000 | Dr. Mark Brown |
| rabbit anti-AKH | 1:1000 | Dr. Mark Brown |
| rabbit anti-DILP2 | 1:2000 | Dr. Jan Veenstra (Veenstra *et al.*, 2008) |
| rabbit anti-PER | 1:1000 | Dr. Charlotte Helfrich-Förster (Stanewsky *et al.*, 1997) |
| mouse nc82 anti-Bruchpilot | 1:50 | Dr. Charlotte Helfrich-Förster (Wagh *et al.*, 2006) |
| rabbit anti-HA | 1:2000 | Cell Signaling Technology |
| ***Secondary antibodies and fluorescent stains*** | | |
| DAPI | 1:1000 |  |
| Hoechst (20mM) | 1:1000 |  |
| Nile red | 1.1000 | Sigma Aldrich |
| Rhodamine-phalloidin | 1:1000 | Thermo Fisher Scientific |
| donkey anti-guinea pig Alexa Fluor ® 488 | 1:1000 | Thermo Fisher Scientific |
| goat anti-chicken Alexa Fluor® 488 | 1:1000 | Thermo Fisher Scientific |
| goat anti-mouse Alexa Fluor® 488 | 1:1000 | Thermo Fisher Scientific |
| donkey anti-goat Alexa Fluor® 488 | 1:1000 | Thermo Fisher Scientific |
| donkey anti-goat Star Green | 1:1000 | Abberior |
| donkey anti-guinea pig Alexa Fluor ® 555 | 1:1000 | Thermo Fisher Scientific |
| donkey anti-rabbit Alexa Fluor ® 555 | 1:1000 | Thermo Fisher Scientific |
| goat anti-mouse Alexa Fluor® 555 | 1:1000 | Thermo Fisher Scientific |
| goat anti-guinea pig Alexa Fluor® 647 | 1:1000 | Thermo Fisher Scientific |
| donkey anti-rabbit Alexa Fluor ® 647 | 1:1000 | Thermo Fisher Scientific |
| donkey anti-mouse Alexa Fluor® 647 | 1:1000 | Thermo Fisher Scientific |
| donkey anti-rat Alexa Fluor® 555 | 1:1000 | Thermo Fisher Scientific |

**Supplemental references**

Hermann-Luibl, C., T. Yoshii, P. R. Senthilan, H. Dircksen and C. Helfrich-Förster (2014). The ion transport peptide is a new functional clock neuropeptide in the fruit fly *Drosophila melanogaster*. *Journal of Neuroscience* **34**(29): 9522-9536.

Park, D., J. A. Veenstra, J. H. Park and P. H. Taghert (2008). Mapping peptidergic cells in Drosophila: where DIMM fits in. *PLoS ONE* **3**(3): e1896.

Stanewsky, R., B. Frisch, C. Brandes, M. J. Hamblen-Coyle, M. Rosbash and J. C. Hall (1997). Temporal and spatial expression patterns of transgenes containing increasing amounts of the *Drosophila* clock gene period and a lacZ reporter: mapping elements of the PER protein involved in circadian cycling. *Journal of Neuroscience* **17**(2): 676-696.

Veenstra, J. A., H. J. Agricola and A. Sellami (2008). Regulatory peptides in fruit fly midgut. *Cell Tissue Res* **334**(3): 499-516.

Wagh, D. A., T. M. Rasse, E. Asan, A. Hofbauer, I. Schwenkert, H. Durrbeck, S. Buchner, M. C. Dabauvalle, M. Schmidt, G. Qin, C. Wichmann, R. Kittel, S. J. Sigrist and E. Buchner (2006). Bruchpilot, a protein with homology to ELKS/CAST, is required for structural integrity and function of synaptic active zones in *Drosophila*. *Neuron* **49**(6): 833-844.
