# Supplementary material for: Anti-diuretic hormone ITP signals via a guanylate cyclase receptor to modulate systemic homeostasis in Drosophila
Source: eLife. 2025 Nov 12;13:RP97043. doi: 10.7554/eLife.97043 (PMC12611267; doi:10.7554/eLife.97043)
Supplement: Supplementary file 3. [file elife-97043-supp3.docx]

Supplementary File 3: Root IDs (v783) of ITPa-producing cells in the FlyWire connectome

| **Neuron type** | **Root ID** | **Hemisphere** |
| --- | --- | --- |
| L-NSC^ITP^ | 720575940630046506 | right |
| L-NSC^ITP^ | 720575940629808911 | right |
| L-NSC^ITP^ | 720575940629872971 | right |
| L-NSC^ITP^ | 720575940627436035 | right |
| L-NSC^ITP^ | 720575940625721118 | left |
| L-NSC^ITP^ | 720575940629924091 | left |
| L-NSC^ITP^ | 720575940631592017 | left |
| L-NSC^ITP^ | 720575940613850262 | left |
| 5^th^-LN_v_ | 720575940619074049 | right |
| 5^th^-LN_v_ | 720575940625254636 | left |
| LN_d_^ITP^ | 720575940627933336 | right |
| LN_d_^ITP^ | 720575940634984800 | left |
